# Evaluation of multiple variate selection methods from a biological perspective: a nutrigenomics case study

**DOI:** 10.1007/s12263-012-0288-4

**Published:** 2012-03-02

**Authors:** Henri S. Tapp, Marijana Radonjic, E. Kate Kemsley, Uwe Thissen

**Affiliations:** 1 Institute of Food Research, Norwich Research Park, Colney Lane, Norwich, NR4 7UA UK; 2 TNO, Microbiology and Systems Biology, P.O. Box 360, 3700 AJ Zeist, The Netherlands; 3 Nutrigenomics Consortium, Top Institute Food and Nutrition, P.O. Box 557, 6700 AN Wageningen, The Netherlands; 4 Present Address: Keygene N.V., P.O. Box 216, 6700 AE Wageningen, The Netherlands

**Keywords:** Multivariate statistical analysis, Nutrigenomics, Microarrays, Pathway analysis, High-fat diet

## Abstract

Genomics-based technologies produce large amounts of data. To interpret the results and identify the most important variates related to phenotypes of interest, various multivariate regression and variate selection methods are used. Although inspected for statistical performance, the relevance of multivariate models in interpreting biological data sets often remains elusive. We compare various multivariate regression and variate selection methods applied to a nutrigenomics data set in terms of performance, utility and biological interpretability. The studied data set comprised hepatic transcriptome (10,072 predictor variates) and plasma protein concentrations [2 dependent variates: Leptin (LEP) and Tissue inhibitor of metalloproteinase 1 (TIMP-1)] collected during a high-fat diet study in ApoE3Leiden mice. The multivariate regression methods used were: partial least squares “PLS”; a genetic algorithm-based multiple linear regression, “GA-MLR”; two least-angle shrinkage methods, “LASSO” and “ELASTIC NET”; and a variant of PLS that uses covariance-based variate selection, “CovProc.” Two methods of ranking the genes for Gene Set Enrichment Analysis (GSEA) were also investigated: either by their correlation with the protein data or by the stability of the PLS regression coefficients. The regression methods performed similarly, with CovProc and GA performing the best and worst, respectively (*R*-squared values based on “double cross-validation” predictions of 0.762 and 0.451 for LEP; and 0.701 and 0.482 for TIMP-1). CovProc, LASSO and ELASTIC NET all produced parsimonious regression models and consistently identified small subsets of variates, with high commonality between the methods. Comparison of the gene ranking approaches found a high degree of agreement, with PLS-based ranking finding fewer significant gene sets. We recommend the use of CovProc for variate selection, in tandem with univariate methods, and the use of correlation-based ranking for GSEA-like pathway analysis methods.

**Electronic supplementary material:**

The online version of this article (doi:10.1007/s12263-012-0288-4) contains supplementary material, which is available to authorized users.

## Introduction

In many life science studies, large data sets are generated from metabolomics, proteomics and transcriptomics experiments. Measurement of numerous relevant metabolites, proteins and genes in a single experiment allows an almost unbiased investigation into the important potential biomarkers or crucial pathways associated with the original study goal. Interpreting the results, however, requires dedicated techniques that can select or rank variates from large amounts of data. Usually, statistical models are generated that describe the relationship between the genomics data and some feature of interest (e.g., a phenotype). The models are then further analyzed to identify the most important variates.

Many variate selection methods are described in the literature. These can differ in their implementation details or in their fundamental statistical principles (Guyon and Elisseeff 2003; Guyon et al. 2006). An ideal variate selection method has principles and parameters that are well-suited to the particular study goal and/or to the data characteristics, although it is not always straightforward to make these choices in advance. Furthermore, even though the statistical principles of a method may be understood, its utility from a biological perspective is often less obvious.

This paper describes the performance of a number of variate selection or ranking techniques, from both a statistical and biological perspective. Representative of quite dissimilar approaches, the statistical methods used are:

- Partial least squares (PLS) regression (Martens and Naes 1989)—a latent vector (LV) approach;
- Genetic algorithm (GA) (Mitchelle 1998)—a global optimization approach, combined with multiple linear regression (MLR);
- LASSO (Tibshirani 1996) and ELASTIC NET (Zou and Hastie 2005)—least-angle shrinkage approaches;
- Covariance procedure (CovProc)—a PLS variant that uses variate selection based on covariance (Reinikainen and Höskuldsson 2003).

In broad terms, these all involve multivariate regression modeling of some kind and the estimation of a few “meta parameters” to summarize the model complexity. We have additionally made comparisons with univariate regression models built from individual genes and the reference protein data.

The methods were applied to quantitative protein measurements and microarray gene expression data obtained from a nutrigenomics case study described in Radonjic et al. (2009). Nutrigenomics investigates molecular relationships between dietary components and genes, proteins and/or metabolites on a large scale. It addresses the question of how nutrition influences gene transcription, protein expression and/or metabolism, with the aim of understanding how dietary factors can affect an individual’s health on a systems level (Müller and Kersten 2003; Afman and Müller 2006; Baccini et al. 2008; Kaput et al. 2010; Evelo et al. 2011). The data used in the present work originate from a large-scale nutritional intervention survey performed in Apolipoprotein E3-Leiden (ApoE3Leiden) transgenic mice (Radonjic et al. 2009). The study examined the time-resolved development of high-fat-induced obesity and related pathologies and used microarrays to obtain genome-wide hepatic gene expression data. These have been used as the predictors in the variate selection methods. We have focused on this single data set to allow a detailed evaluation of the biological relevance of the genes and gene sets selected by the statistical approaches used in this study. Two dependent variates have been considered: plasma concentrations of the proteins Leptin (LEP) and Tissue inhibitor of metalloproteinase 1 (TIMP-1). They were chosen for their relevance to obesity development and inflammation-related tissue remodeling upon high-fat feeding, respectively.

## Materials and methods

### Study design, tissue collection and analysis

A detailed description of the study characteristics including study design, sample preparation, RNA isolation and quality control is described by Radonjic et al. (2009). This section only describes the parts that are relevant for understanding the present work.

The study involved a longitudinal comparison of hepatic gene expression between animals fed a control diet and those fed diets high in either animal or vegetable fat. The mRNA expression levels were determined using NuGO Affymetrix Mouse GeneChip arrays (NuGO_Mm1a520177) and hepatic RNA material from groups of animals from each diet immolated at eight time points (0 days (chow fed), 1 day, 3 days, 1, 2, 4, 8, 12 and 16 weeks) during a 16-week trial. In total, 116 microarray samples were taken for further analysis, comprising 3–6 biological replicates per diet, per time-point. After applying data preprocessing pipeline, hepatic gene expression values were obtained for 15,105 genes with unique identifiers and 73 Affymetrix controls. From a total of 15,178 features, 10,072 were selected based upon the following two criteria: first, for at least one of the diet-time categories, there were two or more absolute expression values greater than a threshold value of 5 units. Second, the maximum-to-minimum expression ratio was >1.5, equivalent to a difference of 0.585 in log_2_ transformed data. Such expression data set has been used as the predictors in the variate selection methods.

In the same high-fat feeding study, plasma concentrations of multiple inflammatory proteins and chemokines were measured with multiplex technologies (Rodent Map v.2.0, Rules Based Medicine, USA). In total, protein data were available for 115 animals. Two proteins (LEP and TIMP-1) were considered as dependant variables for assessing the performance of the variate selection methods evaluated in the current study.

In total, 88 ApoE3Leiden liver and plasma samples were selected from the original study, on the basis of animal-matching data availability for both hepatic transcriptomics and protein measurements for assessing the performance of variate selection methods in the current study. The size of the gene expression matrix analyzed in this study was, therefore, of dimensions [88 × 10,072]. The matching 88 animals included 31 mice fed chow diet, 33 mice fed animal fat diet and 24 mice fed plant fat diet.

### Regression analysis

Multivariate modeling and univariate correlation analysis were performed using Matlab (Mathworks Inc.). The Matlab modeling routines are available on request. The transcriptomics data were log_2_ transformed before analysis, which is a standard step prior to statistical analysis (Van den Berg 2006). All the regression methods used unit variance scaling. Models were assessed by cross-validation using 10 blocks. Single cross-validation (SCV) was used to determine the final model’s meta-parameters, and double cross-validation (DCV) (Smit et al. 2007; Stone 1974) was used to assess predictive performance and model consistency.

*Partial Least Squares (PLS)* Partial least squares is a well-known supervised multivariate latent vector modeling technique (Boulesteix and Strimmer 2007; Martens and Naes 1989). It is not a variate selection method. The number of PLS factors that minimized a modified form of the Amemiya Prediction Criterion, APC, (Norušis and SPSS Inc 1990) was considered to be the optimal meta-parameter: APC(*a*) = [(*n* + *a*)/(*n* − *a*)][1 − (*r*_scv_)^2^], where *n* is the number of observations, *a* is the number of PLS factors used in the model, and *r*_scv_ is the Pearson correlation between the actual values and single cross-validated predictions. The stability of the regression coefficient was calculated by dividing the final SCV coefficients by a jackknife estimate of their standard error, calculated from both DCV and SCV results, as described by Faber (2002). This was used as a basis for ranking the genes for use in conjunction with Gene Set Enrichment Analysis (see below).

*Genetic Algorithm (GA)* Genetic algorithm in combination with multiple linear regression (MLR) was implemented according to Kemsley et al. (2007) and McLeod et al. (2009). The GA is a global optimization variate selection method that builds MLR models based on the best subset of variates. The closest analogue to a meta-parameter is the number of variates used in the final model. GA regression was implemented using an in-house scheme developed at the Institute of Food Research. The GA is a global optimization variate selection method that builds multiple linear regression models based on small subsets of variates. The GA aims to optimize both the model size (number of variates) as well as identifying the best subset. The minimum model size considered was 2 variates, and the maximum size was 69 and 78 for double cross-validation, DCV, and single cross-validation, SCV, respectively. Population sizes of 340 and 308 models were used for the DCV and SCV, respectively. The model fitness criterion used was the mean squared residuals based on block cross-validation. The cross-validation partitions were permuted after each generation. The most successful (fittest) model automatically passed to the next generation. All models in the current population could potentially act as parents although the breading probability was weighted toward the fitter models. The algorithm halts if either of two criteria is met: 30 generations without a change in the fittest model, or if a maximum of ~200 generations has passed. The size of the offspring model is chosen as a randomly assigned number that spans the size range of the parents, with a finite possibility of this value reducing by one. There are three mutation mechanisms: in neighbor and correlation-based annealing, there are finite probabilities of one variate swapping with either an adjacent variate or with one of its five most correlated alternatives. The third mechanism is the possibility of replacing or including a new variate chosen from either the list of all possible variates or from those present in the current population. Duplicate progeny is replaced with immigrants with the same number of variates as the current best model.

*Least absolute shrinkage and selection operator (LASSO)* (Tibshirani 1996) finds regression coefficients that minimize the squared residuals while also being constrained such that the sum of the absolute coefficient values is less than a given value, *t*, which is the meta-parameter. The L1 constraint causes many of the regression coefficients to be set to zero, which makes LASSO a variate selection method. No upper limit was set to the number of variates used in candidate models and the optimum model chosen is that which minimized the Aikake Information Criteria, AIC (Norušis and SPSS Inc 1990).

*Elastic Net* is an extension to LASSO that uses an additional L2 “*ridge*-*regression”* constraint, λ_2_, which is the second meta-parameter to be estimated (Zou and Hastie 2005). This overcomes two limitations of LASSO: (1) the number of selected variates in the model is restricted by the data sample size, and (2) only one variate is selected from a group of highly correlated ones. Candidate models were limited to a maximum of 200 variates.

*Covariance procedure (CovProc)* is a PLS-based variate selection method (Reinikainen and Höskuldsson 2003). The variates are ranked in descending order of the absolute magnitude of the coefficients of the first vector. For variance scaled data, this corresponds to introducing variates based on the strength of correlation with the dependant variate. Regression models were evaluated that used increasing numbers of variates, introduced in five-variate increments, based on the order of the ranked list. The values of the two model meta-parameters (number of variates, number of PLS factors) chosen in the final model corresponded to the combination that resulted in the overall minimum APC.

### Biological interpretation of variate selection results

Ingenuity Pathway Analysis suite (IPA, Ingenuity^®^ Systems. http://www.ingenuity.com, version date May 2009) was used to analyze biological functions of the genes in the final models of CovProc, LASSO, ELASTIC NET and GA, by employing “Overrepresentation analysis” module. Biological function overrepresentation analysis aims to gain a mechanistic insight into the underlying biology of a selected group of genes. It evaluates whether gene sets associated with particular biological functions—such as those represented by Gene Ontology (GO) annotations—are statistically overrepresented in the identified gene group (for example, list of differentially expressed genes or group of genes selected by multivariate models). In this study, Fisher’s exact test *p* values were used to score the significance of biological functions among the genes in the final models of the four appraised variate selection approaches.

Gene Set Enrichment Analysis (GSEA, Subramanian et al. 2005) was used to evaluate the biological relevance of ranking the genes based on two approaches: by their correlation *r* with the protein data and by the stability of the PLS regression coefficients. The ranked gene lists were supplied as inputs into the PreRanked scoring procedure available within the GSEA software. In GSEA, a score is produced, similar to the Kolmogorov–Smirnov statistic, which summarizes the distribution of a predefined set of genes within a prioritized list of genes. Higher scores are given to gene sets that are distributed near the top or bottom of the list. The likelihood of achieving a given score is evaluated by recalculating the score after repeated random permutations of the list order. When multiple gene sets are evaluated, the permutation-based *p* values are used to control the false discovery rate (FDR). Our analysis used gene sets from Molecular signature database (MSigDB) C2 curated gene sets collection (http://www.broad.mit.edu/gsea/msigdb September 2008). A gene set size filter (min = 15, max = 500) removed 737 of the 1,687 gene sets, leaving 950 to be used in the analysis. After collapsing 10,072 native features (gene identifiers from the gene expression data set) into gene symbols, 9,985 genes were recognized and used for the analysis. The number of permutations was set to 1,000. The permutations are used to assign *p* values to the GSEA scores calculated for each gene set. This avoids assuming the scores belong to some underlying distribution. As we evaluated 950 gene sets, the permutation-based *p* values are also used to control the false discovery rate, FDR (e.g., Benjamini and Hochberg 1995). The significantly enriched gene sets referred to in the “Results and discussion” section are those that passed Benjamini and Hochberg FDR threshold: gene sets are considered significantly enriched at false discovery rate (FDR) smaller than 1% (*q* value ≤ 0.01).

The MSigDB service was used to find significant (*p* ≤ 0.01) overlaps between CovProc selected genes and gene sets in the collection.

## Results and discussion

Hepatic genome-wide gene expression levels and plasma protein levels in high-fat diet fed ApoE3Leiden mice were analyzed using five multivariate regression methods: CovProc, LASSO, ELASTIC NET, GA and PLS (“Materials and methods”). The multivariate models were used to prioritize genes that predict the expression of two proteins associated with obesity phenotypes upon high-fat feeding, namely Leptin (LEP) and Tissue inhibitor of metalloproteinase 1 (TIMP-1). This allows elucidation of hepatic molecular mechanisms and the identification of biomarkers associated with deregulated adiposity and tissue remodeling, respectively, observed upon administration of high-fat diets.

### Performance of five multivariate regression methods: model performance

The results of the double cross-validation (DCV) prediction comparison are shown in Table 1. For both proteins studied, CovProc and GA produced the best and worst predictions, respectively, CovProc only slightly exceeding LASSO, and all the variate selection methods performing comparably to PLS. These results can be put into context by considering the correlation *r* between individual genes and the protein data. We find that the numbers of significantly (*p*(*r*) ≤ 0.05/10,072) correlated genes were 208 (2.1% of all genes) and 486 (4.8%) for LEP and TIMP-1, respectively. Single gene regression models were evaluated using single cross-validation (SCV) to allow direct comparison with the results in Table 1. For LEP and TIMP-1, respectively, 18 and 40 genes had an individual predictive ability greater than that obtained by GA; and for TIMP-1, one gene (*Serpina3n*) performed even better than CovProc. This is perhaps a surprising finding, as the widespread use of multivariate analysis (MVA) methods in traditional applications involving high-dimensional data, such as spectroscopy, is due to the improved predictive ability they offer through the “multivariate advantage,” which deals with confounding systematic variation in the predictor data. Our findings imply that—for transcriptomic data, at least—univariate methods should also be investigated.

**Table 1:** Performance of five multivariate regression methods for the prediction of LEP and TIMP-1: predictions using double cross-validation

Method	LEP		TIMP-1	
	*r* ^2^	SEV	*r* ^2^	SEV
LASSO	0.614	1.65	0.698	0.980
ELASTIC NET	0.577	1.75	0.682	0.899
CovProc	**0.762**	1.31	**0.701**	0.864
GA	0.451	2.34	0.482	1.242
PLS	0.621	1.63	0.650	0.925

Bold values indicate the best performance

*r* ^2^, squared correlation between predicted and actual values; SEV, root mean squared residuals

Note that all the variate selection methods could potentially have selected a single variate, and in the case of TIMP-1, this would have led to an improved predictive performance. That all the MVA methods instead selected multiple variates can be interpreted as evidence of overfitting during the model optimization procedure.

The estimated values of the meta-parameters and SCV performance during the 10 rounds of DCV and for the SCV on the whole data set are provided in Online Resource 1.

### Comparison of subset selection methods from a statistical perspective

The genes selected by CovProc, LASSO, ELASTIC NET and GA for LEP and TIMP-1 are summarized in Tables 2 and 3, respectively. Genes present in the final SCV models are emboldened. Also shown is the number of occurrences of each gene during the rounds of DCV and the correlation with each protein. The annotations of these genes can be found in Online Resource 2.

**Table 2:** The genes selected by CovProc, LASSO, ELASTIC NET and GA methods for LEP

CovProc			LASSO			ELASTIC NET			GA		
Gene	*f*	*r*	Gene	*f*	*r*	Gene	*f*	*r*	Gene	*f*	*r*
**Cfd**	10	0.829	**G0s2**	10	0.826	**G0s2**	10	0.826	**G0s2**	2	0.826
**G0s2**	10	0.826	**Cfd**	9	0.829	**Cfd**	9	0.829	**Mogat1**	5	0.816
**Mogat1**	10	0.816	**Mogat1**	9	0.816	**Mogat1**	9	0.816	**D630002G06Rik**	2	0.669
**Omd**	10	0.799	**Cidec**	9	0.770	**Cidec**	8	0.770	**Elovl5**	0	0.604
**Cidea**	10	0.797	**Mme**	8	0.686	**Mme**	8	0.686	**Gstz1**	0	0.582
**Clstn3**	10	0.797	**Gpr98**	7	0.755	**Gpr98**	7	0.755	**Apoa4**	0	0.565
**Aldh3a2**	10	0.784	**Scd1**	7	0.740	**Scd1**	7	0.740	**Perp**	0	0.484
**Cidec**	10	0.770	**Gstk1**	5	0.755	**Gstk1**	5	0.755	**Bloc1s1**	0	0.266
**Gpr98**	10	0.755	Fabp2	4	0.683	Fabp2	4	0.683	**2700050L05Rik**	0	−0.246
**Gstk1**	9	0.755	D630002G06Rik	4	0.669	D630002G06Rik	4	0.669	**Ubxd1**	0	−0.242
**Inhbe**	9	0.751	Omd	3	0.799	Omd	3	0.799	**Ascc3l1**	0	−0.222
**Tnfrsf19**	9	0.744	Pgrmc2	3	0.644	3110032G18Rik	3	0.725	**Eif2a**	0	−0.213
**Scd1**	9	0.740	3110032G18Rik	2	0.725	Pgrmc2	3	0.644	**Lrrc8d**	0	0.212
**Gpc1**	9	0.733	Cidea	1	0.797	Cd36	2	0.727	**Pir**	0	−0.197
**Cd36**	9	0.727	Clstn3	1	0.797	Clstn3	1	0.797	**D2hgdh**	0	0.175
**3110032G18Rik**	9	0.725	Gpc1	1	0.733	Aldh3a2	1	0.784	**9430028L06Rik**	0	0.142
S3-12	4	0.705	Cd36	1	0.727	Gpc1	1	0.733	**Myh9**	0	−0.121
Apom	4	−0.696	EG624219	1	0.593	EG624219	1	0.593	**Zbtb43**	0	0.116
1110028A07Rik	3	0.702	Hectd2	1	0.567	Hectd2	1	0.567	**0610037D15Rik**	1	−0.102
Aqp8	3	0.699	Nnt	1	0.531	Apoa4	1	0.565	**Thnsl2**	0	−0.096
Gbe1	3	0.691	Abcg5	1	0.521	Nnt	1	0.531	**Pitpnm2**	0	−0.085
Mme	3	0.686	Apoc2	1	0.456	Abcg5	1	0.521	**Nt5e**	0	0.066
Sema5b	3	0.679	Aldh3a2	0	0.784	Apoc2	1	0.456	**Il13ra1**	0	−0.059
D630002G06Rik	3	0.669	Inhbe	0	0.751	Cidea	0	0.797	**Bag2**	0	−0.050
Cyp2b9	3	0.659	Tnfrsf19	0	0.744	Inhbe	0	0.751	**Pik3r4**	0	0.046
Fabp2	2	0.683	S3-12	0	0.705	Tnfrsf19	0	0.744	**Xrcc6**	0	−0.040
Vnn1	2	0.679	1110028A07Rik	0	0.702	S3-12	0	0.705	**Sec61a2**	0	−0.012
Cryz	2	0.677	Aqp8	0	0.699	1110028A07Rik	0	0.702	**Tbcc**	0	−0.006
**16**	37		**8**	22		**8**	23		**28**	281	

*f* Number of occurrences in double cross-validation, *r* correlation coefficient of gene expression and LEP data. Genes present in the final single cross-validation model are in bold. The last row gives the number of genes in the final SCV model (bold) and the number selected at least once during DCV

**Table 3:** The genes selected by CovProc, LASSO, ELASTIC NET and GA methods for TIMP-1

CovProc			LASSO			ELASTIC NET			GA		
Gene	*f*	*r*	Gene	*f*	*r*	Gene	*f*	*r*	Gene	*f*	*r*
**Serpina3n**	10	0.862	**Serpina3n**	10	0.862	**Serpina3n**	9	0.862	**Serpina3n**	7	0.862
**Lcn2**	10	0.839	**Apcs**	8	0.807	**Apcs**	7	0.807	**Antxr2**	0	0.581
**Serpina10**	10	0.839	**Cobl**	8	0.781	**Cpb2**	7	0.784	**Keg1**	0	−0.524
**Saa2**	10	0.828	**B3galt1**	8	0.780	**Cobl**	7	0.781	**Cul1**	0	0.462
**Fgl1**	10	0.827	**Cpb2**	6	0.784	**B3galt1**	6	0.780	**Pscdbp**	0	0.414
**Itih4**	10	0.821	**Hapln4**	5	0.709	**Hapln4**	3	0.709	**Ugcgl1**	0	0.401
**Lbp**	10	0.820	Cyb561	3	0.819	Spp1	3	0.631	**LOC634731**	0	0.395
**Cyb561**	10	0.819	**Cxcl1**	3	0.686	**Arl6ip5**	3	0.629	**Ica1**	0	0.367
**Cpne8**	10	0.813	Gm527	3	0.653	**Cxcl1**	2	0.686	**Pilra**	1	0.364
**Apcs**	10	0.807	**2200001I15Rik**	3	0.640	Gm527	2	0.653	**Nudt18**	0	0.338
**Tnfrsf1a**	10	0.805	Spp1	3	0.631	**2200001I15Rik**	2	0.640	**Unk**	1	0.329
**Slc41a2**	9	0.813	**Arl6ip5**	3	0.629	Serpina10	1	0.839	**Cyp17a1**	0	0.299
**Itih3**	9	0.790	Fgl1	2	0.827	Cyb561	1	0.819	**Pdia6**	0	0.296
**Tmem176a**	9	0.785	Acp6	2	−0.554	Tnfrsf1a	1	0.805	**Rap2b**	0	0.248
**Stat3**	9	0.784	Serpina10	1	0.839	Tmem176a	1	0.785	**Pqlc2**	0	0.236
**Cpb2**	9	0.784	Itih4	1	0.821	Mt2	1	0.784	**Tlr8**	0	0.196
**Mt2**	9	0.784	Tnfrsf1a	1	0.805	Abhd14b	1	−0.693	**Tbc1d13**	0	−0.176
**Lrg1**	9	0.783	Tmem176a	1	0.785	Polg2	1	−0.630	**Cxxc5**	0	−0.145
**Cobl**	9	0.781	Litaf	1	0.760	Edg5	1	0.585	**1700006J14Rik**	0	−0.145
**B3galt1**	9	0.780	Abhd14b	1	−0.693	Acp6	1	−0.554	**NA**	0	0.132
Ifitm2	9	0.776	Polg2	1	−0.630	Lcn2	0	0.839	**Itfg1**	0	0.131
**Orm2**	8	0.790	Edg5	1	0.585	Saa2	0	0.828	**Fstl1**	0	0.098
Zbp1	8	0.766	Lcn2	0	0.839	Fgl1	0	0.827	**AI854517**	0	0.081
Orm1	8	0.763	Saa2	0	0.828	Itih4	0	0.821	**Ccdc79**	0	−0.036
Litaf	8	0.760	Lbp	0	0.820	Lbp	0	0.820	**5730410I19Rik**	0	−0.029
Hp	7	0.765	Cpne8	0	0.813	Cpne8	0	0.813	**Dync2li1**	1	0.015
**21**	148		**9**	22		**9**	20		**26**	245	

*f* Number of occurrences in double cross-validation, *r* correlation coefficient of gene with TIMP-1 data. Genes present in the final single cross-validation model are in bold. The last row gives the number of genes in the final SCV model (bold) and the number selected at least once during DCV

*CovProc* As the predictor data were unit variance scaled, genes are introduced by CovProc based on the magnitude of their correlation with the protein. The final models for LEP and TIMP-1 used the first 16 and 21 most correlated genes, respectively. Note that in both cases, all the selected genes had positive values of *r* (i.e., positive correlation). For LEP, all the genes in the SCV model were selected at least 9 times during DCV. For TIMP-1, *Orm2* was the only gene selected in the SCV model not selected at least 9 times during DCV. Similarly, *lfitm2* was the only gene selected at least 9 times during DCV not to be included in the SCV model. As these are only slight differences, we can conclude that both final models were stable.

*LASSO/ELASTIC NET* Tables 2 and 3 show that for both proteins, there was considerable consistency between these methods. Both methods used the same genes in their SCV models. The total numbers of genes selected at least once during DCV were also similar, as were the individual genes: there were 21 common genes selected for LEP and 19 for TIMP-1. This can be attributed to the ELASTIC NET models tending toward relatively small values for the ridge parameter and, therefore, behaving similarly to LASSO (see Online Resource 1). For both proteins, all the genes used in the SCV model had significant values of *r*. There was also agreement in the genes selected by these methods and by CovProc. For LEP, all 8 genes were also present in the 16 gene model selected by CovProc. For TIMP-1, there were 5 genes common to the models by LASSO/ELASTIC NET (9 genes) and CovProc (21 genes) models. This agreement indicates that LASSO and ELASTIC NET preferentially selected genes with high absolute values of *r*. The four genes not present in the TIMP-1 CovProc model were ones less frequently selected during DCV.

*GA* Models selected by the GA showed the least stability—many genes were selected with a frequency, *f*, of just 1. In the interests of conciseness, therefore, the results in Tables 2 and 3 comprise genes selected in the final SCV model ordered by the magnitude of the correlation to each protein. For LEP, *Mogat1* was the most selected during DCV (5 occurrences). For TIMP-1, *Serpina3n* was selected in 7 of the DCV models. This was the most correlated gene and was also selected by the other variate selection methods. Of the genes present in the final SCV model, only 7 and 3 were significantly correlated with LEP and TIMP-1, respectively. A total of 281 and 245 genes were selected at least once during DCV for LEP and TIMP-1, respectively, indicating a lack of consistency in the GA models. Two possible contributing factors for this lack of consistency are first, the large model space—10,072 variates—and thus great potential for converging on local minima; and second, that MLR lacks any mechanism for rejecting noise.

### Evaluation of variate selection methods from a biological perspective

To evaluate the biological relevance of the selected subsets and prioritized lists, the following two-step strategy was used. First, a biological function analysis was used to assess whether a given gene list (SCV final model) or gene ranking was biologically meaningful in terms of the significant gene groups they represent. Second, we considered whether these gene groups were consistent with the physiological role of LEP and TIMP-1.

### Overrepresentation of biological functions for CovProc, LASSO, ELASTIC NET and GA selected genes

The biological relevance of the genes selected by the CovProc, LASSO/ELASTIC NET and GA was assessed using biological function overrepresentation analysis within the Ingenuity Pathway Analysis suite. The results are provided in the Online Resource 3. Based on the *p* value of the biological function category, CovProc performed best with lowest *p* values of 2.43E−06 and 2.33E−07 for LEP and TIMP-1, respectively. LASSO and ELASTIC NET performed similarly with the lowest *p* value of 5.05E−04 for LEP and 1.44E−05 for TIMP-1. GA performed least well, with a lowest *p* value of 1.30E−03 for LEP and 1.15E−03 for TIMP-1. These results are in broad agreement with the regression-based evaluation of these methods.

### Gene Set Enrichment Analysis of *r*- and PLS-ranked gene lists

GSEA of LEP found 40 (22 positively and 18 negatively) significantly enriched gene sets using correlation *r*-based ranking, and 3 (3 positively and 0 negatively) using PLS regression vector stability ranking. For TIMP-1, GSEA found 51 (29 positively and 22 negatively) and 33 (16 positively and 17 negatively) enriched gene sets using the *r*- and PLS-ranked lists, respectively.

**Fig. 1:**
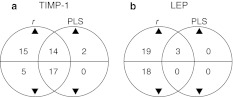
Venn diagrams comparing the numbers of significantly enriched gene sets from GSEA using *r*- and PLS-based ranking for **a** TIMP-1 and **b** LEP. The *arrow* direction depicts whether the comparison concerns numbers of gene sets with positive (*filled triangle*) or negative (*filled inverted triangle*) enrichment

Investigation into the overlaps between gene sets identified by the two ranking approaches found that all (LEP) or most (TIMP-1) of the gene sets identified using PLS ranking were also identified by the *r*-based approach. This was true for both positively and negatively enriched gene sets (Fig. 1a, b). Only two positively enriched gene sets found using PLS ranking for TIMP-1 were not also found using the *r*-based approach.

Interestingly, many gene sets that were found positively enriched in the LEP GSEA results were also negatively enriched in the TIMP-1 results and vice versa (Table 4). This is likely a consequence of the biological roles of these two proteins. TIMP-1 and LEP are associated with inflammation and fat metabolism, respectively, processes perturbed during hepatic response to a high-fat challenge. These responses are conversely timed: inflammation is evoked during the early phase (day 1 to week 2) and repressed during the late phase (week 4 to week 16) of the high-fat diet response, while lipid metabolic adaptations show an opposite temporal pattern and are repressed during early and induced during the late phase of the high-fat feeding time-course (Radonjic et al. 2009). Given the inverse temporal expression of LEP and TIMP-1 under the experimental conditions used in this study (data not shown), it may be expected that gene sets that are positively correlated with the expression of the one protein are negatively correlated with the expression of the other protein.

**Table 4:** Enriched gene sets identified by GSEA using *r*-based ranking

Gene sets	
**ABBUD_LIF_UP (T5) [47]**	▲●
ADIP_VS_PREADIP_DN	▲
AGEING_KIDNEY_SPECIFIC_UP	▲
BASSO_GERMINAL_CENTER_CD40_UP	▲
BROCKE_IL6	▲●
DAVIES_MGUS_MM	▲
IDX_TSA_DN_CLUSTER1	▲
KRETZSCHMAR_IL6_DIFF	▲●
**LEE_DENA_UP (T2) [60]**	▲
LEE_MYC_E2F1_UP	▲
LIAN_MYELOID_DIFF_GRANULE	▲
LIAN_MYELOID_DIFF_RECEPTORS	▲●
LINDSTEDT_DEND_8H_VS_48H_UP	▲
**NI2_MOUSE_UP (T2) [40]**	▲●
RIBAVIRIN_RSV_UP	▲●
ROSS_CBF_MYH	▲
TAKEDA_NUP8_HOXA9_3D_UP	▲
AGEING_KIDNEY_UP	▲$\nabla$
CARIES_PULP_HIGH_UP	▲$\nabla$●
CARIES_PULP_UP	▲$\nabla$●
FLECHNER_KIDNEY_TRANSPLANT_REJECTION_UP	▲$\nabla$●
GALINDO_ACT_UP	▲$\nabla$
**ICHIBA_GVHD (T6) [335]**	▲$\nabla$●
LAL_KO_3MO_UP	▲$\nabla$●
LAL_KO_6MO_UP	▲$\nabla$●
NADLER_OBESITY_UP	▲$\nabla$●
NEMETH_TNF_UP	▲$\nabla$
TARTE_PC	▲$\nabla$●
WIELAND_HEPATITIS_B_INDUCED	▲$\nabla$
AGEING_KIDNEY_SPECIFIC_DN	▼△●
BETA_ALANINE_METABOLISM	▼△●
BUTANOATE_METABOLISM	▼△○●
ELECTRON_TRANSPORT_CHAIN	▼△●
FATTY_ACID_DEGRADATION	▼△
**FATTY_ACID_METABOLISM (L2) [86]**	▼△●
FLECHNER_KIDNEY_TRANSPLANT_REJECTION_DN	▼△●
HUMAN_MITODB_6_2002	▼△●
**IDX_TSA_UP_CLUSTER6 (L2) [166]**	▼△●
KREBS_TCA_CYCLE	▼△○●
LEE_DENA_DN	▼△●
LYSINE_DEGRADATION	▼△
MITOCHONDRIA	▼△●
MOOTHA_VOXPHOS	▼△●
PROPANOATE_METABOLISM	▼△●
VALINE_LEUCINE_AND_ISOLEUCINE_DEGRADATION	▼△○●
BILE_ACID_BIOSYNTHESIS	▼
GLUTATHIONE_METABOLISM	▼●
HCC_SURVIVAL_GOOD_VS_POOR_UP	▼
IDX_TSA_UP_CLUSTER5	▼●
TRYPTOPHAN_METABOLISM	▼●
WANG_MLL_CBP_VS_GMP_DN	▼
**ADIP_VS_FIBRO_UP (L3) [35]**	△
**ADIP_VS_PREADIP_UP (L3) [36]**	△
**LEE_CIP_UP (L4) [62]**	△
LEE_MYC_TGFA_DN	△
**TNFALPHA_ADIP_DN (L2) [59]**	△
**ZMPSTE24_KO_DN (L2) [32]**	△
IDX_TSA_DN_CLUSTER2	$\nabla$
MYOD_NIH3T3_DN	$\nabla$
ROS_MOUSE_AORTA_DN	$\nabla$
STEMCELL_COMMON_DN	$\nabla$
TRANSLATION_FACTORS	$\nabla$
TRNA_SYNTHETASES	$\nabla$

*Key to symbols* positively (▲) and negatively (▼) enriched gene sets found for TIMP-1; positively (△) and negatively ($\nabla$) enriched gene sets found for LEP; gene sets also found using PLS-based ranking for TIMP-1(●) and LEP (○). Emboldened gene sets were also identified from the CovProc selected variates. The size of the gene set is given in square brackets, and the number of CovProc identified genes present for TIMP-1 (T) or LEP (L) is shown in round brackets

### Relevance of biological analysis results in the context of LEP and TIMP-1 functions

Measurements of plasma protein concentrations of Leptin (LEP) and Tissue inhibitor of metalloproteinase 1 (TIMP-1) were considered as two dependent variates for the analysis in this study. These proteins were chosen due to their relevance in addressing the following research question: What are the processes underlying onset and progression of metabolic disorders (such as obesity) associated with high-fat feeding? The early hepatic effect of high-fat feeding involves induction of inflammatory and immune processes, while the late adaptation to excess dietary fat results in hepatic fat accumulation and development of hepatic steatosis (Radonjic et al. 2009). A statistically significant association between circulating plasma parameters and these hepatic physiological processes may be employed for the development of noninvasive diagnostics of the systemic disorder caused by high-fat feeding. To specifically target the representatives of inflammatory and adipogenic processes, we selected TIMP-1 and LEP plasma protein levels from the pool of plasma parameters that were assessed in the high-fat feeding study (Radonjic et al. 2009).

LEP is a circulating adipocytokine that regulates fat mass in response to nutritional status. It plays an important role in maintaining energy homeostasis and metabolic rate and its plasma levels are affected by energy-rich nutrients such as fatty acids, carbohydrates and proteins (Ahima and Flier 2000; Zou and Shao 2008). In agreement with the physiological role of LEP, the most significant functional category identified by the analysis of genes in the CovProc final SCV model is related to lipid metabolism (Online Resource 3). Also with high significance (*p* = 4.75E−04), was the category “carbohydrate metabolism.” Lipid and carbohydrate metabolism were also represented in LASSO/ELASTIC NET (*p* = 3.03E−03) and GA results (*p* = 1.30E−03 to 7.77E−03). Additionally, the GA model identified genes involved in metabolism of amino acids/proteins. Consistent with the role of LEP, GSEA found significant positively enriched gene sets related to amino acid metabolism, fatty acid metabolism, energy yielding processes such as oxidative phosphorylation and tricarboxylic acid (TCA) cycle, and conditions associated with increased adiposity (Table 4). In the context of using subset selection methods (CovProc, LASSO/ELASTIC NET and GA) to find markers associated with a given biological parameter, *GOs2*, *Cfd* and *Mogat1* could be considered as the top three markers associated with LEP. They were selected by all the final models, and all have functions associated with lipid metabolism. Specifically, GOS2 regulates adipose lipolysis; CFD (adipsin) is involved in systemic lipid metabolism or energy balance; and MOGAT1 catalyzes the synthesis of precursors of physiologically important lipids such as triacylglycerol and phospholipids (Cook et al. 1987, Yen et al. 2003, Yang et al. 2010). Regarding the crucial role of LEP in energy homeostasis, lipid metabolism and liver pathophysiology, the specific processes mediated by GOS2, CFD and MOGAT1 may suggest the possible routes via which LEP accomplishes these functions.

TIMP-1 has a role in the degradation of extracellular matrix proteins in response to various stimuli in both normal and pathological conditions including morphogenesis, tissue repair, tumorigenesis and cell death (Gaudin et al. 2000; Guedez et al. 1996; Ray and Stetler-Stevenson 1994). Additionally, TIMP-1 is produced by lymphocytes as an important factor in facilitating leukocyte infiltration into inflammatory sites during inflammatory response (Johnatty et al. 1997). In agreement with the roles of TIMP-1, the most significant functional category identified by the CovProc SCV final model is related to “inflammatory response” (*p* = 2.33E−07) (see Online Resource 3). The category “Hepatic System Disease” is also found significant among CovProc results (1.75E−04). Similarly, the category “inflammatory response” is also highly significant among LASSO and ELASTIC NET results (*p* = 1.44E−05). The GA method performed less well, with *p* value of 1.72E−02 for the same category. The significant positively enriched gene sets identified by GSEA of TIMP-1 are associated with several pathological states, including inflammation-related pathologies, tissue rejection during transplantation, hepatomas, hepatitis and disorders caused by inflammatory agents (Table 4). The overlap of significant gene sets with Gene Ontology categories (The Gene Ontology Consortium 2000) reveals that “immune system process” and “inflammatory response” are the most relevant biological processes underlying the above listed pathologies (*p* value 4.58E−9 and 2.82E−7, respectively, for the significance of the overlap with the most significant gene set). For TIMP-1, *Serpina3n* was selected as the top-ranked associated gene in all the final models (CovProc, LASSO/ELASTIC NET and GA) and can, therefore, be considered as the most relevant marker. SERPINA3N is a protease inhibitor, and deficiency of this protein has been linked to liver disease. A direct functional link between TIMP-1 and SERPINA3N has not been established yet, but from their cellular roles, it is likely that they act interdependently in degrading the extracellular matrix proteins during inflammatory response and/or other conditions.

Considering the functions of LEP and TIMP-1, we may conclude that all methods performed well in the identification of biologically relevant genes.

In summary, CovProc was the best performing MVA subset selection method. Similarly, for GSEA, the *r*-based ranking performed better than the ranking based on the stability of the PLS regression coefficients. In terms of biological relevance, the choice between these two methods will depend on the research goal. While CovProc will be more suitable for selecting a limited set of markers associated with a given dependent parameter, GSEA using *r*-based ranking may provide a more global insight into biological processes related to this parameter.

### Direct comparison of CovProc selected variates with pathways prioritized by the ranking methods

To directly compare CovProc selected variates with pathways prioritized by the ranking methods, the 16 and 21 genes used in the final SCV CovProc models for LEP and TIMP-1, respectively (bold in Tables 2 and 3), were overlapped with the total C2 gene sets collection (1,892 gene sets including 17,544 genes). Using a *p* value threshold of 0.01, 15 gene sets were identified for LEP and 9 for TIMP-1.

Of the identified gene sets, 7 and 4 were also identified by *r*-ranked GSEA and 0 and 3 identified by PLS-ranked GSEA for LEP and TIMP-1, respectively (Table 4). This shows that the biological interpretation of genes selected by CovProc corresponds well with the interpretation of the *r*-ranked results. All the overlapping gene sets between *r* and CovProc are found among positively enriched gene sets. This is consistent with CovProc selected genes that were exclusively positively correlated with LEP and TIMP-1.

## Conclusions

This study has compared five methods currently used for variate selection or ranking: PLS, GA, LASSO/ELASTIC NET and CovProc. Based on statistical model performance and parsimony, the GA is outperformed by the other methods, with CovProc as the best method. From a biological perspective, it appears that all methods select meaningful variates, either for variate subsets (CovProc, LASSO/ELASTIC NET) or for gene rankings (correlation and PLS coefficient stability), although CovProc somewhat outperforms the other methods for selecting a definite list of genes. We would also recommend that any multivariate analysis should be used in conjunction with more traditional univariate analyses. The results of biological interpretation using *r*-based rankings are superior to those using ranking by PLS coefficient stability.

In conclusion, based on the biological interpretability of the results, CovProc and correlation-ranked methods are both highly recommended, complementary methods for analyzing transcriptomic data. CovProc is particularly suited to select a limited set of markers associated with a given biological parameter, while correlation-ranked GSEA is more appropriate for gaining global insight into biological processes associated with that parameter.

## Electronic supplementary material

Below is the link to the electronic supplementary material.

The estimated values of the meta-parameters and SCV performance during the 10 rounds of DCV and for the SCV on the whole data set (PDF 78 kb)

The annotations of genes selected by the SCV models of CovProc, LASSO, ELASTIC NET and GA for LEP and TIMP-1, listed in Tables 2 and 3 of the accompanying manuscript (XLS 71 kb)

The results of biological function overrepresentation analysis (Ingenuity Pathway Analysis suite) demonstrating biological relevance of the genes selected by the CovProc, LASSO/ELASTIC NET and GA final models (bold in Table 2 and 3) (XLS 231 kb)
