# Supplementary material for: Evaluation of multiple variate selection methods from a biological perspective: a nutrigenomics case study
Source: Genes Nutr. 2012 Mar 2;7(3):387–97. doi: 10.1007/s12263-012-0288-4 (PMC3380194; doi:10.1007/s12263-012-0288-4)
Supplement: Supplementary file 1 — The estimated values of the meta-parameters and SCV performance during the 10 rounds of DCV and for the SCV on the whole data set (PDF 78 kb) [file 12263_2012_288_MOESM1_ESM.pdf]

Journal: Genes and Nutrition

Evaluation of multiple variate selection methods from a biological perspective: a nutrigenomics case study

Henri S. Tapp[#], Marijana Radonjic[#] E. Kate Kemsley and Uwe Thissen

Corresponding author:

Marijana Radonjic, TNO, Microbiology and Systems Biology P.O. Box 360, 3700 AJ Zeist, The Netherlands **marijana.radonjic@tno.nl**,

Online Resource 1.

Estimated optimum values of meta parameters associated with each of the five methods for leptin and TIMP-1 from single cross-validation during each of the 10 rounds of double cross validation (9 CV partitions) and during the final single-cross validation (10 CV partitions): t, sum of absolute regression coefficients; nvars, number of variates; sep, root mean squared residual from single CV; λ_2, ridge coefficient; npls, number of PLS factors.

	LASSO			*ELASTIC NET*				*CovProc*			*GA*		*PLS*	
CV block	t	nvars	sep	t	nvars	λ_2	sep	nvars	npls	sep	nvars	sep	npls	sep
<u>leptin</u>														
1	2.85	11	1.26	2.95	11	0.037	1.26	16	1	1.03	28	0.57	4	1.59
2	2.57	12	1.36	3.50	13	0.367	1.21	26	2	0.95	28	1.12	3	1.70
3	1.95	7	1.17	2.79	7	0.592	0.93	21	1	0.80	25	0.74	3	1.37
4	2.22	8	1.28	2.28	8	0.024	1.28	11	1	0.93	29	1.75	4	1.59
5	2.65	11	1.34	2.67	11	0.008	1.33	16	1	1.05	32	0.57	3	1.71
6	1.35	7	1.59	1.35	7	0	1.59	26	1	1.01	25	0.93	3	1.65
7	2.34	9	1.39	2.59	10	0.114	1.37	16	1	1.05	29	0.85	3	1.70
8	0.54	4	1.83	0.66	3	0.466	1.80	26	1	1.01	45	1.30	4	1.65
9	2.40	8	1.32	2.70	9	0.128	1.29	16	1	1.04	30	1.32	5	1.93
10	2.60	12	1.28	2.60	12	0	1.28	26	1	0.99	25	0.53	3	1.60
Single	2.25	8	1.29	2.53	8	0.128	1.26	16	1	1.00	28	0.63	4	1.59
<u>TIMP-1</u>														
1	1.25	11	0.86	1.34	11	0.073	0.85	101	4	0.71	20	0.51	3	0.90
2	0.93	5	0.92	1.19	5	0.283	0.87	11	1	0.78	23	0.42	2	0.93
3	0.95	6	0.94	1.36	4	0.789	0.88	131	7	0.72	23	0.41	2	0.93
4	1.31	12	0.81	1.37	12	0.051	0.80	31	1	0.71	22	0.54	2	0.85
5	0.60	4	1.04	1.05	1	10	0.92	106	1	0.73	8	0.73	2	0.91
6	1.26	8	0.87	1.38	8	0.184	0.86	26	1	0.79	25	0.61	2	0.94
7	1.13	7	0.93	1.42	5	0.438	0.88	31	1	0.79	23	0.71	2	0.95
8	1.05	6	0.97	1.37	5	0.522	0.90	36	1	0.80	55	1.07	3	0.92
9	1.07	8	0.93	1.37	8	0.395	0.88	21	1	0.80	29	0.92	1	1.03
10	1.02	8	0.86	1.31	1	32.5	0.78	36	1	0.68	27	0.44	3	0.78
Single	1.16	9	0.88	1.24	9	0.069	0.86	21	1	0.76	26	0.46	2	0.90
